# The H^+^-ATPase (V-ATPase): from proton pump to signaling complex in health and disease

**DOI:** 10.1152/ajpcell.00442.2020

**Published:** 2020-12-16

**Authors:** Amity F. Eaton, Maria Merkulova, Dennis Brown

**Affiliations:** Program in Membrane Biology and Division of Nephrology, Massachusetts General Hospital and Harvard Medical School, Boston, Massachusetts

**Keywords:** acidification, endosomal trafficking, pathophysiology, pH regulation, proton pumping ATPase

## Abstract

A primary function of the H^+^-ATPase (or V-ATPase) is to create an electrochemical proton gradient across eukaryotic cell membranes, which energizes fundamental cellular processes. Its activity allows for the acidification of intracellular vesicles and organelles, which is necessary for many essential cell biological events to occur. In addition, many specialized cell types in various organ systems such as the kidney, bone, male reproductive tract, inner ear, olfactory mucosa, and more, use plasma membrane V-ATPases to perform specific activities that depend on extracellular acidification. It is, however, increasingly apparent that V-ATPases are central players in many normal and pathophysiological processes that directly influence human health in many different and sometimes unexpected ways. These include cancer, neurodegenerative diseases, diabetes, and sensory perception, as well as energy and nutrient-sensing functions within cells. This review first covers the well-established role of the V-ATPase as a transmembrane proton pump in the plasma membrane and intracellular vesicles and outlines factors contributing to its physiological regulation in different cell types. This is followed by a discussion of the more recently emerging unconventional roles for the V-ATPase, such as its role as a protein interaction hub involved in cell signaling, and the (patho)physiological implications of these interactions. Finally, the central importance of endosomal acidification and V-ATPase activity on viral infection will be discussed in the context of the current COVID-19 pandemic.

## INTRODUCTION

**Figure 1.**
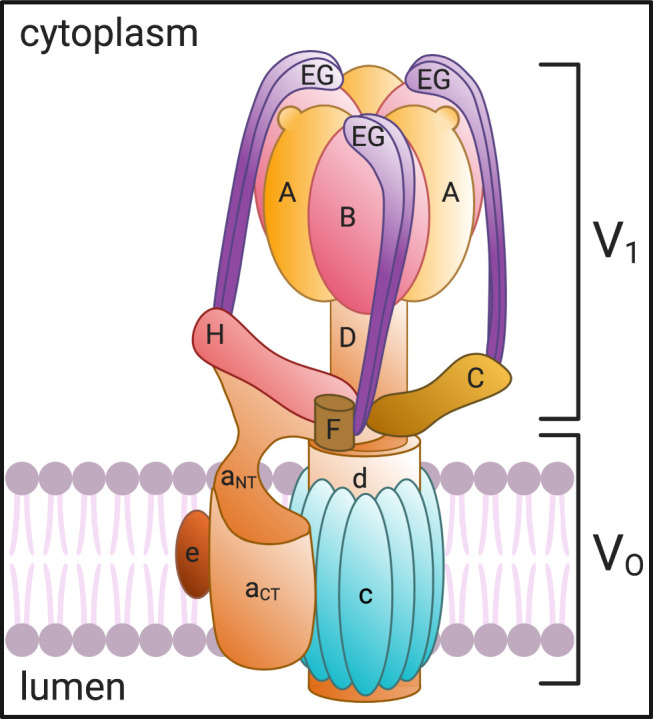
V-ATPase structure and subunit composition. The V-ATPase is composed of a transmembrane (V_O_) domain comprising the a, c, d, and e subunits and a cytosolic (V_1_) domain made up of the A, B, C, D, E, F, G, and H subunits. ATP is hydrolyzed at the intersection of the A and B subunits, which powers the rotation of the rotor formed by the d, D, and F subunits. The “c-ring” couples the energy generated by ATP hydrolysis to the translocation of protons from the cytosol to the lumen through the hemichannel formed between the a subunit and the proteolipid c-ring. Created with BioRender.com.

It has been appreciated for decades that a primary function of the H^+^-ATPase (or V-ATPase) is to create an electrochemical proton gradient across eukaryotic cell membranes, which energizes fundamental cellular processes. Initially characterized in the yeast vacuole (hence, V-ATPase), its activity notably allows for the acidification of intracellular vesicles and organelles, which is necessary for many essential cell biological events to occur (1–5). Preventing intracellular compartmental acidification by disrupting the gene encoding the critical proteolipid c-ring (see below, and Fig. 1) of the V-ATPase is embryonic lethal (6), highlighting the important contribution of the V-ATPase to cellular function. Other V-ATPase subunits were also identified as essential for viability in a broad genetic screen in mice (7). However, this screen may not be perfect; it identified knockout of the ATP6Voa4 subunit as lethal, but at least two viable a4 knockout (KO) mouse lines have been made and used in physiological studies on V-ATPase function (8, 9). Furthermore, inhibiting V-ATPase activity with one of several drugs, most commonly bafilomycin or concanamycin, also has serious, deleterious effects on cells (10–13), although targeted V-ATPase inhibition is a potential therapy in cancer and perhaps some other disease conditions, such as osteoporosis (see below).

**Figure 2.**
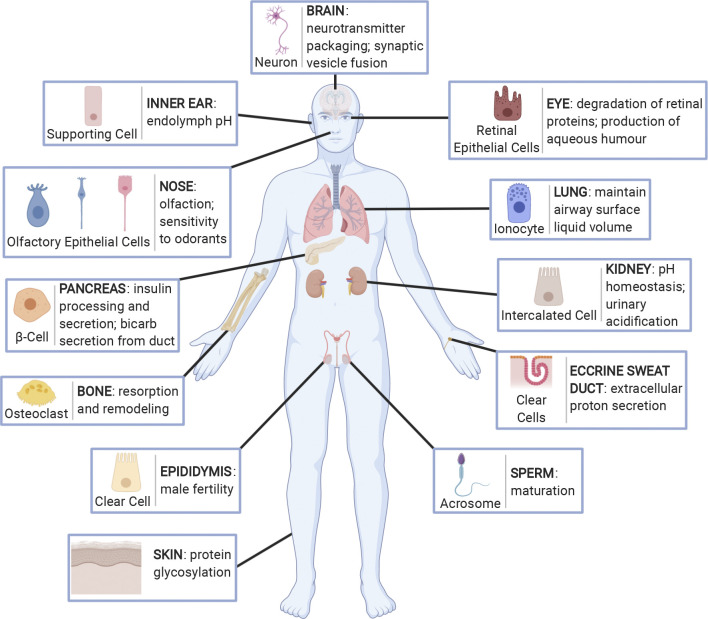
Organs and tissues in which V-ATPase membrane expression and proton secretion play a major role in physiological function. Specific V-ATPase holoenzymes are expressed mostly at the apical surface of specialized proton-secreting cells in several tissues throughout the body. These include the supporting cells and interdental cells in the inner ear, epithelial cells in the olfactory mucosa of the nose, β-cells in islets of Langerhans and secretory duct cells in the pancreas, osteoclasts in bone, clear cells in the epididymis, retinal pigment epithelial cells in the eye, ionocytes in the lung, kidney intercalated cells, clear cells in eccrine sweat ducts, and the acrosome of sperm cells. Furthermore, the V-ATPase is expressed ubiquitously in the endomembrane system of cells where it acts to acidify intracellular vesicles. In this capacity the V-ATPase plays particular roles in the brain, for example, where it is involved in the packaging and release of neurotransmitters, as well as soluble *N*-ethylmaleimide-sensitive factor attachment protein receptor (SNARE)-dependent membrane fusion of synaptic vesicles, and in the skin where it is involved in proper protein glycosylation. Created with BioRender.com.

In addition to its participation in what might be called “housekeeping” functions within the cell, many specialized cell types in various organ systems such as the kidney, bone, male reproductive tract, inner ear, olfactory mucosa, and more, use plasma membrane V-ATPases to perform specific activities that depend on extracellular acidification (Fig. 2). Finally, and importantly, it is increasingly apparent that V-ATPases are central players in other normal and pathophysiological processes that directly contribute to human health in many different and sometimes unexpected ways. The first part of this review will cover the well-established role of the V-ATPase as a transmembrane proton pump in the plasma membrane and intracellular vesicles, followed by a discussion of the more recently emerging unconventional roles for the V-ATPase, such as its role as a protein interaction hub involved in cell signaling, and the (patho)physiological implications of these interactions. However, first, it is informative to understand the molecular composition and organization of this unusually complicated enzyme because its variable and complex subunit structure provides multiple avenues by which organelle- and cell-specific functions can be regulated.

## MOLECULAR ORGANIZATION OF THE V-ATPase

The eukaryotic vacuolar V-ATPase is an evolutionarily conserved, nanoscale proton-pumping rotary motor whose composition and organization closely resemble the mitochondrial ATP synthase, or F_o_F_1_ ATPase; both were derived from a common ancestral enzyme (14). Whereas the ATP synthase uses a transmembrane proton gradient to generate ATP, the V-ATPase uses the energy of ATP hydrolysis to create a proton gradient across various biological membranes. The V-ATPase is a multiprotein complex composed of 14 subunits, which form two distinct domains (Fig. 1). The transmembrane V_O_ sector generally has five different subunits, which form the pore allowing for the transmembrane passage of protons, while the cytosolic V_1_ sector harnesses the energy of ATP hydrolysis to power the rotation of the motor and drive protons through the transmembrane V_O_ pore (2). Although the structure and function of the V-ATPase are highly conserved across eukaryotes, there are multiple isoforms and splice variants of most of the V-ATPase subunits that differ by species and that show tissue, cell, and subcellular specificity in their expression patterns (15). This variability was initially described in yeast in which two distinct isoforms of the “a” subunit, Vps1p and Stv1p, are responsible for targeting the V-ATPase to the vacuole or the Golgi and endosomes, respectively (16). Similarly, in mammalian cells, four different isoforms of the transmembrane “a” subunit also direct the V-ATPase to distinct cellular sites (15, 17). Many of the numerous mammalian splice variants were identified by the screening of large online genomic datasets, and their in vivo expression and potential relevance to cell- and membrane-specific location and function of the holoenzyme have not yet been established (18). The characterization of these subunit variants in terms of specific patterns of protein expression and function could, therefore, be a fruitful area of future research that would contribute to our understanding of differential cell expression, regulation, and function of the V-ATPase in different cells. For example, while the four common isoforms of the transmembrane “a” subunit are known as a_1–4_, a total of 11 splice variants were actually identified in our database search, raising the need for further work to dissect the cellular roles of this important V_O_ sector component (18). This is overshadowed, however, by the remarkable discovery that 17 “a” subunit genes are present in *Paramecium*, providing a rich tapestry of potential V-ATPase assemblies to direct intracellular targeting and function (19).

## MICROSCOPY OF THE V-ATPase

**Figure 3.**
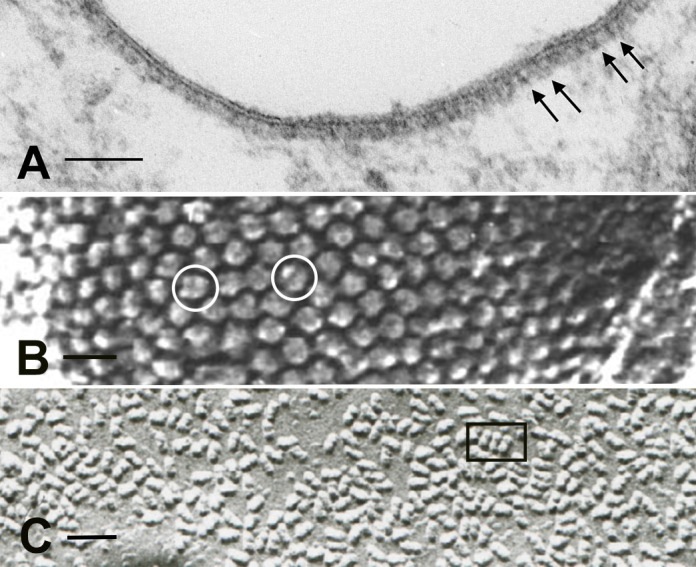
Microscopy of the V-ATPase in proton-secreting cells. The large cytoplasmic V_1_ domain of the V-ATPase is clearly detectable as an electron dense array of coating material attached to the underside of the lipid bilayer in a kidney intercalated cell apical plasma membrane (*A*; arrows; bar = 0.1 µm). The rapid-freeze, deep etching procedure reveals the dense, hexagonally packed arrays of V_1_ domain stud-like projections attached to a vesicle in a proton-secreting cell from toad urinary bladder (*B*; circles; bar = 25 nm). The freeze fracture technique, exposing the internal domain of split-open lipid bilayers, reveals numerous so-called rod-shaped or dumbbell-shaped (e. g., inside the rectangle) intramembranous particles in V-ATPase-rich membranes: this image is from a kidney intercalated cell (*C*; bar = 50 nm).

The V-ATPase holoenzyme is an extremely large protein complex of ∼830,000 Da and is readily visible by conventional and freeze-etching electron microscopy (EM) (Fig. 3, *A* and *B*). It also lends itself to cryo-EM studies at the nanoscale level, resulting in the definition of its exquisite structural complexity (20). The large, electron dense, membrane-associated V_1_ domains, first noticed in transmission EM images of insect epithelia, were called portasomes by Harvey and colleagues (21) to indicate a probable role in ion (most likely K^+^) transport (they were, in fact, recognized as being morphologically related to the mitochondrial F_o_F_1_ ATPase) (22). Subsequently, morphological studies demonstrated that the presence of similar structures on the apical and/or basolateral membrane of turtle bladder and intercalated cells was correlated with their vectorial proton-secreting ability (23–25).

The “portasomes” were definitively identified as V-ATPase molecules in toad bladder proton-secreting cells (26) using the rapid-freeze, deep-etch microscopy technique. The structures were packed into hexagonally arranged, paracrystalline arrays on the cytosolic side of the membrane, at a maximum density of over 10,000 molecules per square micrometer (Fig. 3*B*). They were found on both the apical plasma membrane and associated with cytoplasmic vesicles, consistent with a recycling mechanism described below. Importantly, V-ATPase molecules isolated from bovine kidney medulla, and incorporated into phospholipid vesicles in the absence of any other proteins, formed identical hexagonal arrays (26). Similar structures were subsequently identified by the deep-etch procedure on the contractile vacuole of *Dictyostelium*, which is responsible for osmoregulation in many single celled organisms (27). Anti-V-ATPase antibodies applied to kidney intercalated cells using immunogold electron microscopy confirmed that these structures were indeed the cytoplasmic domains of the proton pump (26), and subsequent studies confirmed that the V-ATPase was also present in membranes from insect transporting epithelia (28, 29).

An interesting morphological feature also associated with many membranes that contain large numbers of V-ATPase molecules is the presence of so-called “rod-shaped” intramembranous particles, as visualized by freeze-fracture electron microscopy. With this technique, the interior of frozen lipid bilayers is exposed and then visualized by making a platinum/carbon replica. By EM, distinct particles are seen in the replicas, representing the transmembrane domains of proteins (30). While most membrane proteins appear as globular structures usually between 5–12 nm in diameter, V-ATPase-rich membranes contain characteristic elongated particles ∼20-nm long that frequently appear to be composed of two (or sometimes more) globular particles fused together, often resulting in a dumbbell like appearance (Fig. 3*C*). Such particles are commonly seen in V-ATPase-rich intercalated cells in the kidney, amphibian epidermis and bladder, turtle bladder, osteoclasts, and narrow cells in the epididymis (31). They are found both on the plasma membrane and on intracellular vesicles, and their distribution mirrors the presence of V-ATPase “studs” seen by conventional EM (see above). However, while they correlate with V-ATPase in some membranes (32), their exact identity remains a mystery. Their dumbbell shape implies that they are at least dimeric in nature. They are certainly formed by the transmembrane domains of proteins and, thus, may represent the “a” or “c” subunits of the V_O_ sector of the pump, but they are not found in all membranes that contain the V-ATPase, even at quite high levels, such as plasma membranes of kidney proximal tubule epithelial cells (33) and epididymal clear cells (34), suggesting that either they represent a protein closely associated with the V-ATPase in some but not all membranes or that they are associated with a specific subunit composition of the V-ATPase. Unfortunately, with only a small number of laboratories currently performing the highly specialized technique of freeze-fracture microcopy, it is unlikely that the identity of these enigmatic particles will be revealed anytime soon.

## REGULATION OF V-ATPase ACTIVITY

Transmembrane proton transport by the V-ATPase can be regulated in several different ways to modify pH in extracellular compartments or within intracellular vesicles.

### Reversible Assembly and Disassembly

The V-ATPase is an example of a protein complex in which some of the components (V_O_) have transmembrane domains and are, thus, synthesized in the rough endoplasmic reticulum, whereas others are totally cytosolic (V_1_) and are produced on “free” ribosomes. These two domains must, therefore, associate at some point to produce a functional V_1_V_O_ holoenzyme. This provides an opportunity for regulation by modulating the efficiency and extent of the association process to stimulate or inhibit proton pumping activity across a given membrane. This phenomenon was first described in yeast where the V_1_ domain reversibly disassociates from the membrane-bound V_O_ domain in response to glucose deprivation and a variety of other cellular signals. This is believed to be a physiological “starvation” response aimed at reducing energy utilization in the absence of glucose (35). Importantly, on their own, the separated domains are inactive, with protons unable to pass through V_O_ while not bound to V_1_ (36, 37) and with V_1_ lacking ATPase activity in the absence of V_O_ (38). This presumably ensures that nonassociated V_O_ domains will not serve as proton-leak pathways across membranes and that V_1_ domains will not hydrolyze ATP in a futile cycle in the absence of the holoenzyme complex. However, the purified transmembrane c-ring of the yeast enzyme has been reported to act as a conductance pore under some conditions (39). Other examples of the use of reversible assembly to regulate proton secretion are in the insect midgut (40) and in blowfly salivary glands (41, 42). There are only a few clear examples of assembly/disassembly regulating V-ATPase activity in mammalian cells in vivo, although some reports presented data supporting a yeast-like, glucose-dependent regulation of this process in cells in culture, while another study demonstrated reversible assembly on synaptic vesicles (43–45). Increased vesicle acidification along the endosomal pathway has also been correlated with the amount of assembled holoenzyme in the various vesicular fractions of cultured baby hamster kidney cells (46), and increased V-ATPase assembly on lysosomes has been reported during dendritic cell maturation (47). Finally, amino acid starvation leads to V-ATPase assembly on lysosomes (48), as discussed in more detail below.

Several regulatory proteins involved in its biosynthesis and assembly associate with the V-ATPase in yeast. Some of the best characterized form a complex known as the regulator of ATPase of vacuoles and endosomes (RAVE) complex (Rav1, Rav2, and Skp1), which is required for the biosynthesis and assembly of functional Vps1-containing V-ATPase complexes at the vacuolar membrane (49). Intriguingly, the RAVE complex does not regulate the assembly of Stv1p-containing V-ATPase complexes suggesting that, in addition to the information encoded in the “a” subunit that distinguishes these enzymes, the expression of specific accessory proteins is also involved in targeting of the V-ATPase to distinct membranes. More recently, the mammalian homologues of Rav1, known as rabconnectins, were identified and shown to regulate V-ATPase activity (50). Hinting at their essential function, many of these newly described accessory proteins associate with the V-ATPase as strongly or even more strongly than some of the V-ATPase subunits themselves (51). Of note, some of these mammalian V-ATPase accessory proteins appear to have unique tissue and subcellular expression, which suggests they may play a role in differential V-ATPase targeting and function in mammalian cells, as in yeast, and this area is one that requires attention in future studies.

The precise mechanism by which the holoenzyme assembles and disassembles remains poorly understood, but the V_1_ subunit C is believed to play a central role in the interaction between the two domains, at least in part via an interaction with the RAVE complex in yeast (52). Interestingly, a recent study has shown that the glucose-dependent recruitment of RAVE to the yeast vacuolar membrane requires the presence of the V_O_ sector of Vph1 but not the V_1_ sector, raising additional questions about the dynamics of the assembly process (53).

### Regulated Trafficking of the V-ATPase

**Figure 4.**
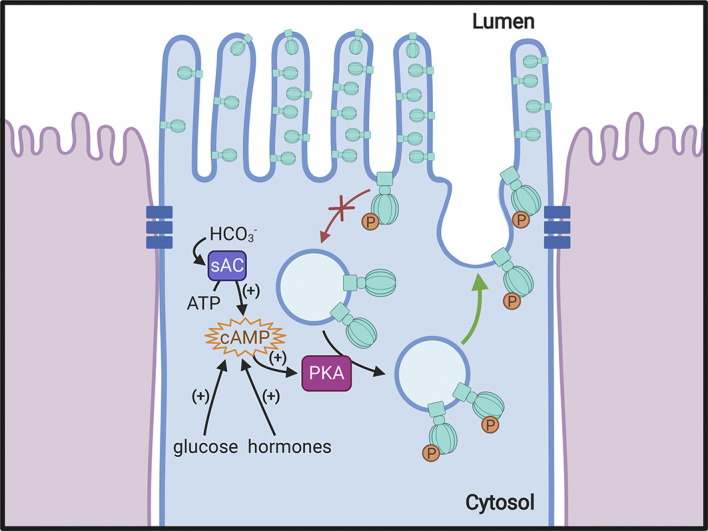
Regulation of V-ATPase by vesicle recycling. Recycling of the V-ATPase between intracellular vesicles and the plasma membrane is a mechanism by which proton secretion is modulated. Incorporation of V-ATPase into the apical membrane occurs in response to various physiological stimuli, including several hormones and glucose. One common feature of the process in different cell types is the activation of PKA by increased intracellular cAMP, resulting in phosphorylation (P) of some V-ATPase subunits, including A and C in the cytosolic V_1_ sector. By an unknown mechanism, this leads to an alteration of the balance between exocytosis (stimulated: green arrow) and endocytosis (reduced: red arrow) of V-ATPase-rich vesicles, resulting in a net accumulation of plasma membrane V-ATPase. One potential stimulus in the kidney and in the epididymis is bicarbonate, generated either by direct entry into the cell through apical bicarbonate exchangers or by the activity of carbonic anhydrase type II in the cytosol. The increased ${\text{HCO}}_{3}^{{}^{-}}$ concentration stimulates the production of cAMP by soluble adenylyl cyclase (sAC), thereby activating PKA. Phosphorylated V-ATPase complexes are shown at a larger size for clarity. Created with BioRender.com.

**Figure 5.**
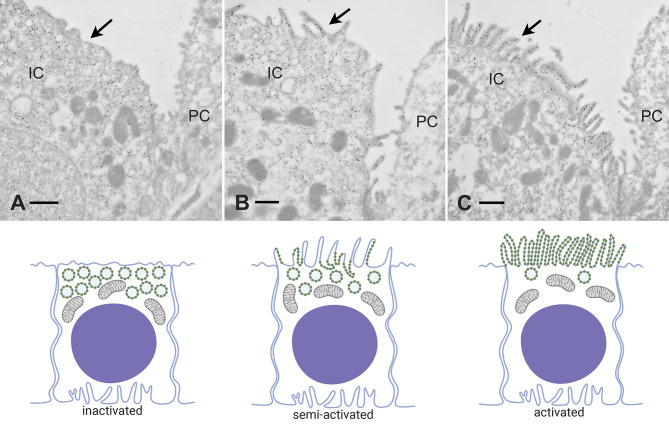
Different “activation” states of kidney intercalated cells. This plate illustrates regulation of acid secretion by shuttling/recycling of V-ATPase between intracellular vesicles and the plasma membrane. *Top*: electron micrographs of intercalated cells (IC) from mouse kidney collecting ducts in different states of activation, using immunogold staining with anti-V-ATPase antibodies. The *Bottom*: schematic representations of the distribution of V-ATPase molecules, vesicles, and apical microvilli that are illustrated at *top*. *A*: “nonactivated” cell in which most of the V-ATPase is concentrated in many intracellular vesicles, with very little apical membrane expression (arrow). *B*: “partially activated” cell in which the V-ATPase is present both in intracellular vesicles, as well as some apical membrane expression coupled with the presence of a few apical microvilli (arrow). *C*: highly “activated” cell in which most of the V-ATPase is concentrated in extensive apical microvilli and microplicae (arrow), with very little remaining inside the cell on cytoplasmic vesicles. PC, principal cell. Bar = 0.5 µm. (Thank you to Drs. Jennifer Pluznick and Nathan Zaidman, Dept. Physiology, Johns Hopkins Univ. School of Medicine for permission to use EM images prepared as part of an ongoing collaboration.)

A second mechanism of controlling V-ATPase activity is via regulated trafficking of the functional holoenzyme (Figs. 4 and 5). This occurs primarily in acid-secreting cells in a variety of different tissues (5, 23). In proton-secreting intercalated cells in the kidney collecting duct and analogous organs in lower vertebrates, such as the turtle and toad urinary bladders and amphibian epidermis, regulation of transepithelial proton secretion is achieved by the exo- and endocytotic recycling of tubulovesicular structures containing high levels of V-ATPase holoenzymes in their membranes (23) (Figs. 4 and 5). Osteoclasts in bone (54) and proton-secreting cells in the epididymis (55) also use this mechanism to control proton secretion acutely. In this way, the amount of V-ATPase incorporated in the plasma membrane is increased or reduced depending on prevailing physiological cues, and proton secretion is modified accordingly (Fig. 5). It is likely that assembly/disassembly and trafficking occur simultaneously in cells, and more work is needed to address the relationship between these two regulatory mechanisms. A close relationship between these two events occurs during synaptic vesicle trafficking, for example, where V-ATPase disassembly appears to be required before synaptic vesicle fusion with the plasma membrane (43). One simplistic explanation for this could be that fusion of the lipid bilayers would otherwise be inhibited by the physical presence of the large V_1_ domain of the pump, especially in acid-secreting cells such as intercalated cells that express large numbers of pumps on each vesicle. Thus more efficient membrane fusion may occur in regions in which the holoenzyme has been disassembled. Some studies have indeed implicated the V_O_ sector in membrane fusion, but this hypothesis remains controversial (43, 56).

The trafficking and recycling process (Fig. 4) involves several associated regulatory proteins, such as kinases and proteases, although relatively few of them have been clearly identified. In addition, a novel G protein-coupled receptor known as Gpr116 was shown to negatively regulate V-ATPase surface expression in kidney intercalated cells; its knockout actually results in an increase in plasma membrane V-ATPase expression, with a concomitant reduction in urine pH reflecting increased proton secretion by these cells (57). The actin and microtubule cytoskeleton is also critically involved in the recycling of V-ATPases, similar to its role in trafficking of many other membrane proteins in most cells. V-ATPase B and C subunits associate with actin (58), and a profilin-like binding site has been identified on both the B1 and B2 subunits (59). Earlier work showed that the microtubule-disrupting drugs colchicine and vinblastine inhibit CO_2_-induced proton secretion by the turtle urinary bladder (60) and disruption of microtubules results in defective trafficking of the V-ATPase in proton-secreting cells, such as intercalated cells (61) and epididymal clear cells (62). Interestingly, V-ATPase disassembly induced by glucose depletion also requires intact microtubules, at least in yeast (63). The process of V-ATPase exocytosis involves interaction with the SNARE (soluble *N*-ethylmaleimide-sensitive factor attachment protein receptor) machinery, as is true for many exocytotic events in other cells (62, 64–66), but in contrast, its endocytotic retrieval occurs via vesicles that do not contain any of the traditional endocytic machinery proteins such as clathrin or caveolin (67, 68). Despite the fact that this clathrin-independent process was first described over three decades ago (69), it is still largely overlooked even in recent reviews on endocytosis (70). Thus the mechanism underlying the nonclathrin, noncaveolin-mediated internalization of the V-ATPase in proton-secreting cells remains an interesting area for future investigation that may also shed light on similar mechanisms involving the V-ATPase in other cells.

It is known that an increase in cellular cAMP as a result of several cues, including hormonal stimulation (23), activates protein kinase A, which can phosphorylate the A subunit and the C subunit of the V-ATPase (Fig. 4). The former appears to be necessary for increased V-ATPase expression at the cell surface (71, 72), while the latter has been implicated in V-ATPase assembly in insect cells (42). Activation of PKA can also occur via the bicarbonate-stimulated soluble adenylyl cyclase, whose regulatory role in acid-base sensing and the activation of proton-secretion has been discussed elsewhere (Fig. 4) (73, 74). Furthermore, AMP kinase (AMPK) also phosphorylates the V-ATPase subunit A and regulates its trafficking in renal epithelial cells (75). The complex interaction between AMPK and the V-ATPase related to aspects of cellular nutrient homeostasis is discussed in more detail below. However, many other putative phosphorylation consensus sites have been identified by proteomic analysis on a variety of other V-ATPase subunits (76). This opens the way for further regulation of function, but very little is known about the role of these additional sites or about the kinases and phosphatases involved in their potential modification. Thus the regulation of the V-ATPase by phosphorylation is a fertile area for future research and may be important to understand the many different patterns of expression and regulation of V-ATPase activity in a variety of cells and tissues, as well as its pathophysiological dysfunction leading to human disease.

## V-ATPase, pH SENSING, AND THE ACIDIFICATION OF INTRACELLULAR VESICLES AND ORGANELLES

It is well known that V-ATPase-mediated acidification of several intracellular compartments is necessary for their biological function. This includes dissociation of receptor-ligand complexes as part of the receptor downregulation and recycling process; proper sequential targeting of vesicles and proteins in the endocytotic pathway of cells; generating an acidic pH in lysosomes to allow optimal activity of acid hydrolases; the loading of neurotransmitters and hormone precursors, such as norepinephrine and proinsulin, into synaptic vesicles and secretory granules; and proper posttranslational modification of proteins in the trans-Golgi network (77–86). All of these processes are disrupted following V-ATPase inhibition or by dissipating the pH gradient using a variety of drugs and other experimental maneuvers. Furthermore, it should also be noted that establishment and maintenance of a defined pH within vesicles depend on the presence of other ion channels and transporters in addition to the V-ATPase itself, including but not limited to Cl^−^ channels, Na^+^/H^+^ and K^+^/H^+^ exchangers, as well as to membrane proton permeability (87).

### pH Sensing

The role of V-ATPase dependent acidification in directing intracellular vesicle traffic is a particularly intriguing phenomenon. The central question is how the intravesicular pH is recognized by the vesicle transporting and sorting machinery, which for the most part comprises cytosolic proteins that are separated from the pH gradient by a lipid bilayer. The first clue was the discovery that some small GTPases were recruited to the vesicle membrane in an acidification-dependent manner to direct the targeting process (88, 89). Subsequently we showed that the V-ATPase itself was able to associate with some of these small GTPases and regulatory proteins in an intravesicular pH-dependent manner (90). This led to the idea that the V-ATPase is not only involved in generating the acidic pH but also in sensing intravesicular pH and transmitting this information to its cytoplasmic domain, enabling trafficking molecules to bind and perform their targeting functions. Many questions still remain unanswered, and while both the transmembrane c and a subunits are believed to be involved, the mechanism by which they undergo modification on their cytosolic domains in response to a luminal acidic pH is unclear. It is possible that luminal histidine groups are part of the pH sensor and that their prevailing charge determines the conformational state of the cytoplasmic domain of V-ATPase subunits that allows “trafficking” proteins to associate with the vesicle. Identifying the endosomal pH sensor is a fruitful area for future research that has repercussions for normal cellular function and for understanding the many pathophysiological processes in which cellular trafficking events are dysfunctional.

### Protein Glycosylation and Viral Uptake

An area of considerable current interest is the role of acidification in protein glycosylation as it relates to coronavirus binding and uptake into cells. The idea that modifying intracellular vesicle acidification might inhibit viral entry is not new, and endosomal acidification is known to play a key role in allowing viral material to enter the cytoplasm of the host cell (91). Work on cultured lung cells during the first severe acute respiratory syndrome (SARS) epidemic suggested that chloroquine effectively reduced viral entry into cultured cells (92). When the mechanism was examined, aberrant terminal glycosylation of the angiotensin-converting enzyme 2 (ACE2) protein, the putative receptor of the viral spike protein at the cell surface, was identified. This modification reduced the affinity of ACE2 for the viral protein, thus restricting binding and reducing viral entry. Following a period of extreme interest in the potential use of hydroxychloroquine in COVID-19 therapy (93), its use is no longer recommended due to the failure of clinical trials to show any positive effect. Nonetheless, targeting V-ATPase mediated acidification remains a valid option for antiviral therapy, and the search for clinically useful and safe drugs for this purpose is ongoing.

## V-ATPase EXPRESSION, PROTON SECRETION, AND ORGAN FUNCTION

In addition to being expressed within intracellular membranes, the V-ATPase is found in the plasma membrane of specialized acid-secreting cells in various tissues and organs (Fig. 2). Here, the V-ATPase’s traditional proton-pumping role drives protons across the plasma membrane and into the extracellular environment. This process plays a critical role in the function of several organs and is acutely regulated in specialized cells by vesicular transport to and from the cell surface and intracellular vesicles, as described above (Figs. 4 and 5).

### Amphibian Epithelia

Much of our understanding of V-ATPase regulation by vesicle trafficking is derived from earlier work on tissues from lower vertebrates. Toad and turtle bladder epithelia, as well as amphibian epidermis, were widely used as models for the kidney collecting duct, since they contain cells known originally as “mitochondria-rich cells” that closely resemble kidney intercalated cells (31), as well as principal cells that undergo vasopressin-stimulated increases in water permeability. A striking feature of these proton-secreting cells that was identified almost 50 yr ago is their high expression of carbonic anhydrase (CA) compared with the surrounding epithelial cells (94). Cytoplasmic CAII, in particular, plays a crucial role in generating the protons that are subsequently transported by the V-ATPase (31). The role of mitochondria-rich cells that are scattered among “principal” cells in these epithelia is associated not only with acid/base regulation but also with the need to energize cellular ion transport processes in a low sodium environment (95). An analogous but more complex situation exists in fish gills, in which V-ATPase-rich ionocytes use V-ATPase to energize Na^+^ and Cl^−^ absorption or secretion in fresh and salt water, respectively (96). We also identified V-ATPase-rich cells in lung epithelium several years ago (97), and more recent data implicate them in CFTR-mediated chloride and ultimately fluid transport in this tissue (98, 99).

**Figure 6.**
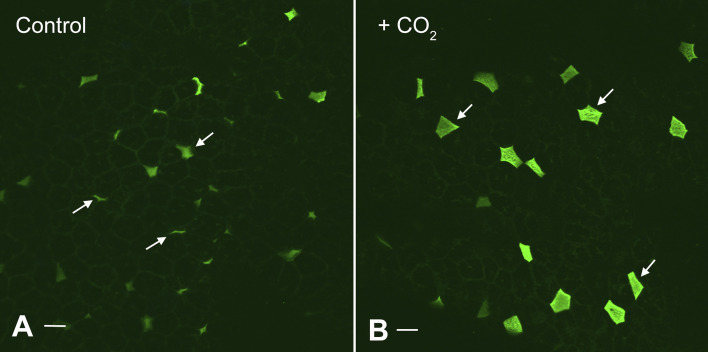
Effect of CO_2_ exposure on expression of V-ATPase and apical membrane area of proton-secreting cells in the turtle urinary bladder. Image shows a whole mount view of the apical epithelial surface of a turtle urinary bladder stained with antibodies against the “A” subunit of the V-ATPase, followed by secondary donkey anti-rabbit Ig coupled to Alexafluor 488. The V-ATPase-rich cells (arrows) show a remarkable increase in surface area between the control (*A*) and tissues acutely exposed to basolateral CO_2_ (*B*), indicating that CO_2_ rapidly activates these cells. Tissue was kindly provided by Dr. John Schwartz, Boston University Medical Center, Boston who exposed bladders to CO_2_ as part of the annual “Origins of Renal Physiology” course at Mount Desert Island Biological Laboratories, Bar Harbor, ME. Bar = 10 µm.

Some of the first evidence showing that V-ATPases could be translocated from vesicles to the apical membrane of proton-secreting cells was generated in turtle bladder upon stimulation with basolateral CO_2_ (100, 101). Electron microscopy was used to follow the cellular location of the characteristic stud-like projections (V-ATPases) in these cells, and electrophysiology was used to measure acid secretion that occurred in parallel with the morphological change (25). This work was done before the generation of anti-V-ATPase antibodies, but we recently applied such antibodies to turtle bladders before and after exposure to CO_2_. As seen in Fig. 6, the positive cells from the stimulated bladder are brighter and have a much greater surface area, consistent with increased V-ATPase-mediated proton secretion. In contrast, during inhibition of proton transport using the disulfonic stilbene SITS in early studies, a reduction in cell surface area was seen using scanning EM and carbonic anhydrase histochemistry (102).

### Kidney Intercalated Cells

**Figure 7.**
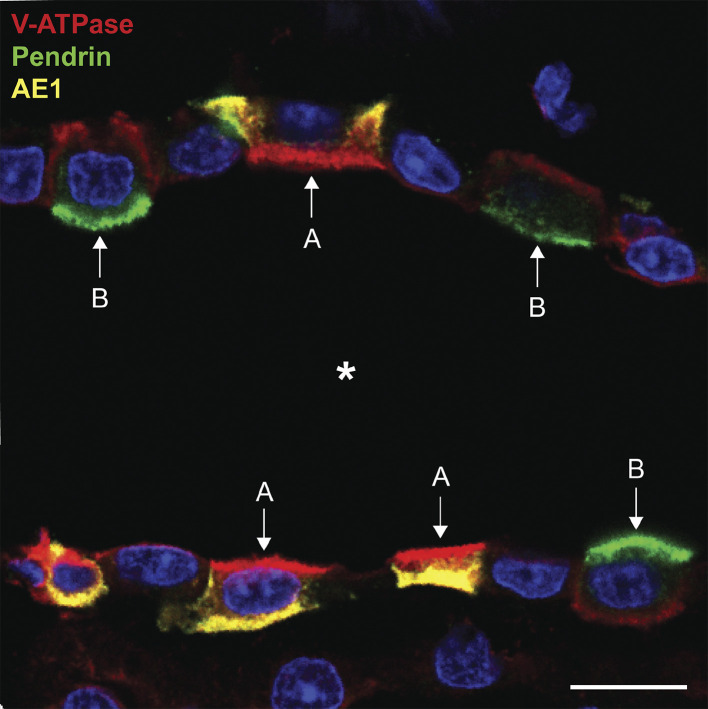
Immunostaining showing type A- and type B-intercalated cells in cortical collecting duct of a rat kidney. Antibodies against the V-ATPase (A-subunit: red), the AE1 anion exchanger (SLC4A1 - yellow), and pendrin (SLC26A4: green) show differential distribution in A- and B-intercalated cells. In this collecting duct, type A cells have apical V-ATPase and basolateral AE1, whereas type B cells have apical pendrin and basolateral V-ATPase. The intensity of basolateral V-ATPase staining in B-cells is lower than the amount of apical staining in the A-cell. Bar = 10 µm. *Tubule lumen.

These highly specialized cells have received considerable attention over the past few decades. Their specific role in maintaining systemic acid/base homeostasis is achieved by shuttling V-ATPase to and from the plasma membrane in response to a variety of physiological cues (Fig. 5) (103–105). One fascinating feature of these cells is that different subtypes in the kidney can either transport protons into the tubule lumen (A-type IC) or into the peritubular space (B-type IC). This is achieved by regulating the polarity of functionally important acid base transporters in their plasma membrane (106–108). There is still debate about the relationship between A- and B-ICs, and how, or even whether, they can rapidly inter-convert depending on prevailing systemic acid-base conditions. One intriguing aspect of these different phenotypes is that the polarized expression of the V-ATPase holoenzyme can be so variable in different intercalated cells. In general, the V-ATPase in A-type cells is distributed between intracellular vesicles and the apical plasma membrane. These cells, as illustrated in Fig. 7, are characterized by the basolateral expression of the Cl^−^/${\text{HCO}}_{3}^{{}^{-}}$ exchanger anion exchanger 1 (AE1), and AE1-positive cells never express V-ATPase alongside AE1 in the basolateral membrane (109). B-type cells, on the other hand, express a different anion exchanger, pendrin, in their apical membrane, and they lack AE1 (110). Pendrin-positive cells (which are restricted to the cortex and, to a much lesser extent, the outer stripe of the outer medulla) can express V-ATPase not only in intracellular vesicles but also to different degrees in their apical and/or basolateral membranes (111). Cells that express apical V-ATPase, but no basolateral AE1, were initially categorized as non-A, non-B cells (112), and subsequently they were shown to also express apical pendrin alongside the V-ATPase (110). It is intriguing that while both type A- and B-intercalated cells are found in cortical collecting ducts, only A-cells populate collecting ducts in most of the outer and inner medulla. As far as we are aware, B-cells have never been found in medullary collecting ducts (other than the very initial portion in the outer stripe of the outer medulla) in adult animals. The developmental and/or environmental factors that are responsible for this location-specific distribution have not so far been identified.

From the viewpoint of V-ATPase biology, a major unresolved question is how does the V-ATPase accumulate in different membrane domains in these cells? What signals the holoenzyme to accumulate basolaterally only in B-IC and never in A-IC? As far as we know at this time, the subunit composition of the apical and basolateral ATPases in these cells is identical. However, the identification of different subunit splice variants, and the lack of reagents to distinguish among these variants, present the possibility that an as yet unknown targeting motif or signal exists to direct the enzyme apically or basolaterally in different cells. Such a signal would probably be contained in the transmembrane V_O_ domain, which could then “attract” the corresponding cytosolic V_1_ domain to the appropriate region of the cell. Indeed, such a targeting role has been shown for the large transmembrane “a” subunit (113), of which 4 isoforms are known but for which a total of 11 splice variants have been identified (18). On the other hand, A- and B-ICs express different cohorts of proteins (114), including the V-ATPase interacting scaffolding protein NHERF1 in B-cells (115), which could play a role in differential protein localization. A major difference between the B1 and B2 isoforms of V-ATPase is the presence of a COOH-terminal PDZ protein binding motif (DTAL) on the B1 but not B2 subunit, but the importance of this difference to V-ATPase localization and function and is unknown.

## NONTRADITIONAL ROLES OF THE V-ATPase

More recently it has emerged that in addition to its traditional proton-pumping role the V-ATPase has many unconventional roles where it forms direct protein-protein interactions involved in signaling pathways, such as during amino acid sensing along the mammalian target of rapamycin complex 1 (mTORC1) pathway (116) or in Wnt and Notch signaling that is involved in cancer tumorigenesis (117). Given these insights it is perhaps not surprising that V-ATPase dysfunction leads to numerous and varied pathologies including diabetes, cancer metastasis, and neurodegenerative diseases (3, 118).

## CANCER

The involvement of the V-ATPase in cancer tumorigenesis has long been known, with unusually high V-ATPase activity promoting cancer and metastasis; inhibition of V-ATPase activity has been shown to inhibit cancer progression (119). Indeed, certain V-ATPase subunits are inappropriately upregulated in a variety of cancer cell lines and tumors and V-ATPase activity is correlated with the invasiveness of cancer cells (120). Furthermore, unconventional V-ATPase complexes expressed at the plasma membrane of cancerous cells enable them to maintain an alkaline cytoplasm despite generating an abnormally acidic extracellular environment (120, 121). This extracellular acidification promotes invasiveness in part by driving proteolytic degradation of the extracellular matrix by enzymes that are optimally active at low pH, such as cathepsins and matrix metalloproteases (122–124). In some cases, the acidic external environment also contributes to drug resistance (125), for example, in osteosarcoma cells (126). Therefore, targeting the mechanisms by which cancer cells generate and survive in acidic microenvironments represents a possible strategy for limiting cancer metastasis. Some studies have even discussed injection of bicarbonate solution into the cancerous mass, or ingestion of sodium bicarbonate, to alkalinize the tumor environment, thereby reducing local acidosis and inhibiting metastasis (127, 128).

Intriguingly, the irregular V-ATPase complexes found at the plasma membrane of highly invasive cancer cells express a unique “fingerprint” of a subunit isoforms (119). The a3 subunit localizes to the leading edge of metastatic breast cancer cells, its expression is much higher in tumor cells than normal cells, and within cancer cells the a3 expression level correlates with invasiveness (129, 130). Furthermore, overexpressing the a3 subunit increases the invasiveness of breast cancer cells in culture, while silencing a3 expression decreases invasiveness (120). These findings suggest that expression of V-ATPase complexes with unique subunit isoform conformations could allow the V-ATPase to function in very different ways depending on the physiological or pathological context. Thus targeting specific a subunit isoforms that are expressed in invasive cells could provide therapeutic potential in treating cancer metastasis.

### Notch Signaling

**Figure 8.**
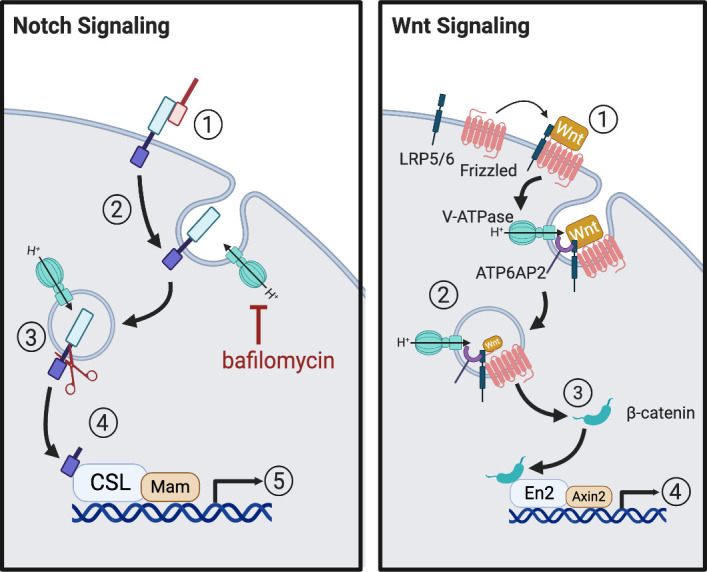
Notch/Wnt signaling pathways. Notch signaling is initiated by ligand binding to Notch receptor (*1*) leading to receptor endocytosis (*2*). The receptor is cleaved by γ-secretase in a V-ATPase-dependent manner (*3*) allowing the cytoplasmic domain to translocate to the nucleus (*4*) and activate target genes (*5*). Inhibition of V-ATPase-mediated acidification with bafilomycin blocks this process. Furthermore, V-ATPase-dependent lysosomal degradation of the receptor decreases Notch signaling. Wnt signaling is initiated by Wnt ligand binding to the Frizzled and low-density lipoprotein (LRP) receptors (*1*) leading to internalization of the receptors into V-ATPase-containing endosomes where they associate with the V-ATPase via the ATP6AP2 accessory protein (*2*). A multiprotein signaling pathway then inhibits a “destruction complex” that normally degrades β-catenin, leading to an increase in cytoplasmic accumulation and translocation of β-catenin to the nucleus (*3*), where it activates target genes (*4*). Created with BioRender.com.

In addition to its traditional role as a proton pump, the V-ATPase is directly involved in regulating the proliferative and inflammatory signaling pathways that are characteristically misregulated in tumor cells (Fig. 8). The Notch signaling pathway controls the balance between cell proliferation and apoptosis, and under normal conditions, activating Notch signaling acts as a tumor suppressor; therefore, decreased Notch signaling is a hallmark of many cancers (131, 132). Activation of the Notch pathway first requires ligand binding, which stimulates receptor endocytosis. Once internalized the receptor is cleaved by γ-secretase and the intracellular portion translocates to the nucleus where it acts a transcription factor related to cellular proliferation. Ultimately the receptor is degraded in the lysosome, dampening the signal (133). In the last decade, it has emerged that activation of Notch signaling requires V-ATPase activity (134). Perhaps it is not surprising that Notch signaling is regulated by V-ATPase activity, given that γ-secretase is optimally active at low pH (135). Furthermore, inhibiting V-ATPase activity using bafilomycin prevents the cleavage and activation of the Notch receptor (50, 134, 136, 137).

In *Drosophila*, zebrafish, and mammalian cells, V-ATPase-dependent Notch activation requires two novel V-ATPase regulatory proteins, Rabconnectin-3A and 3B (Rbcn-3A/3B) (50, 137). Depletion of either prevents the proper acidification of intracellular compartments and disrupts Notch signaling before the cleavage of the intracellular domain, supporting a role for Rbcn-3 in the regulation of V-ATPase-dependent Notch signaling (50, 137, 138). Intriguingly, the yeast homolog of Rbcn-3, Rav1p, is part of the RAVE complex, which regulates V-ATPase activity via reversible assembly of the holoenzyme (139). In mammals there are no known homologs of the other components of the RAVE complex, but on synaptic vesicles Rbcn-3A (Dmxl2) forms a complex with Rbcn-3B (WDR7), which binds Rab3A and participates in neurotransmitter secretion via exocytosis (140).

In addition to Rbcn-3A (Dmxl2), in mammals there is a second rabconnectin-3 isoform Dmxl1, which also shares partial homology to Rav1p. Compared with Rbcn-3A (Dmxl2), Dmxl1 is more highly expressed in the kidney than the brain. We showed that it strongly associates with the V-ATPase B1 subunit and regulates V-ATPase-dependent acidification of intracellular vesicles in cultured kidney cells (51). Additionally, depletion of Dmxl1 using siRNA strongly inhibits the ability of acidic intracellular vesicles to reacidify after bafilomycin treatment and subsequent washout (51). More work will be required to understand how rabconnectins regulate V-ATPase activity in mammals, but the tissue-specific expression of these proteins offers interesting possibilities as therapeutic targets that could provide more specificity than inhibition of the V-ATPase itself. Furthermore, as yet unidentified mutations in some of these accessory “assembly” proteins may be responsible for some human “acidification” diseases for which no candidate genes for the usual suspect proteins (notably the V-ATPase subunits themselves, anion exchangers or carbonic anhydrase isoforms) have yet been recognized.

### Wnt Signaling

The Wnt signaling pathway is also regulated by the V-ATPase and is correlated with the metastasis of cancer cells (117). Wnt signaling is initiated by Wnt ligand binding to the Frizzled and low-density lipoprotein (LRP) receptors, which results in the cytoplasmic accumulation and subsequent translocation of β-catenin to the nucleus where it activates oncogenes (Fig. 8). In frogs and flies, the V-ATPase interacts with the LRP 5/6 receptor complex indirectly via the V-ATPase accessory protein ATP6AP2 and pharmacological inhibition of V-ATPase inhibits Wnt signaling (117, 141, 142). Therefore, disrupting the interaction between the V-ATPase and the LRP receptor complex could potentially block the activation of oncogenic genes via Wnt signaling.

### Immune Signaling

The V-ATPase appears to play a direct role in the aberrant immune signaling that contributes to the inflammatory characteristics of the tumor microenvironment. In cancer cells, the NH_2_ terminus of the a2 isoform of the V-ATPase is cleaved. The resulting peptide plays a proinflammatory role by promoting the transition of macrophages into a pro-oncogenic tumor-associated phenotype and upregulating proinflammatory cytokines (143). Accordingly, knocking down a2 with shRNA in a breast cancer cell line prevented the transition of macrophages to the proinflammatory state and ultimately slowed tumor growth (144). Again, this highlights the therapeutic potential for targeting specific a subunit isoforms to limit cancer metastasis.

### Proton Pump Inhibitors as Cancer Therapeutics

In view of the potential role of the V-ATPase and acidification in cancer progression and metastasis, a number of studies have examined the role of proton pump inhibitors (PPIs) in various forms of cancer. Because it has been used and tested previously for toxicity in humans, the PPI omeprazole has been the most commonly tested drug. While it is best known as an inhibitor of the structurally distinct gastric proton pump (H^+^/K^+^-ATPase), it also affects the V-ATPase at high enough concentrations (145–147). Interestingly, we found several years ago that the V-ATPase in isolated endosomes from kidney cortex was much more sensitive to omeprazole than the V-ATPase in medullary vesicles (148). While not proven, one explanation is that the bulk of the cortical endosomes are derived from proximal tubules, which express the ubiquitous B2 isoform of the B subunit, whereas endosomes from the medulla are enriched in intercalated cell vesicles that contain the more restricted B1 subunit. The inhibitor sensitivity of holoenzymes that express these different isoforms remains to be tested in more detail but could be relevant to informing therapeutic regimes in which cells expressing these distinct subunits are targeted.

## DIABETES

One of the primary ways that cells adapt to changes in glucose availability is via the glucose-dependent assembly of V-ATPase holoenzymes, which in turn modulates V-ATPase function (44, 53, 149–151). In addition to the intrinsic glucose-sensitivity of the V-ATPase assembly state, the V-ATPase controls glucose transport and availability by directly regulating the uptake and secretion of insulin. The primary insulin-sensitive glucose transporter Glut4 and the V-ATPase colocalize on vesicular membranes in adipocytes, a primary target of insulin signaling (152). Furthermore, treatment of adipocytes with bafilomycin inhibited insulin uptake into Glut4-positive vesicles (152, 153). Given its role in glucose signaling and insulin secretion, it is perhaps not surprising that V-ATPase dysfunction has been linked to diabetes. To this point, recent studies have shown increased apical V-ATPase expression and activity in the proximal tubule epithelial cells of diabetic rats, together with a decrease in insulin-containing secretory granules and insulin secretion by the pancreas. Furthermore, intraperitoneal injection of these diabetic rats with bafilomycin increased insulin secretion and reduced plasma glucose levels (154).

Particular V-ATPase complexes, with a similar makeup to the holoenzymes that are responsible for bone remodeling in osteoclasts, are highly expressed in endocrine tissues, including in β-cells within pancreatic islets (153, 155). V-ATPase complexes containing the a3 subunit isoform localize to the plasma membrane of insulin-containing secretory vesicles, and animal studies have demonstrated this isoform regulates insulin exocytosis (153, 156). Oc/oc mice, which contain a null mutation within the a3 gene, present with decreased levels of plasma insulin and show impaired secretion of insulin in response to glucose, despite the functional production and packaging of insulin into partially acidified secretory granules (156). Interestingly, oc/oc mice show increased expression of alternate a subunit isoforms, such as a2, which colocalizes partially with insulin in mutant β-cells, unlike in wild-type mice (156). This raises the possibility that insulin processing and secretory granule acidification, which may normally be mediated by the a3 subunit, are partially compensated for by the increased expression of the a2 isoform. Intriguingly, BHC9 cells, a pancreatic β-cell line, treated with bafilomycin A did not have acidified secretory granules but still maintained insulin secretion, suggesting that V-ATPase expression, but not activity, is required for insulin secretion (156).

### Role of ATP6AP2 in Diabetes

Another key player in the V-ATPase response to glucose is the accessory protein ATP6AP2, otherwise known as the prorenin receptor (PRR), although since receiving this name it has been appreciated that ATP6AP2 plays little role in regulating the renin-angiotensin signaling axis (157). ATP6AP2 regulates biogenesis and assembly of the V-ATPase holoenzyme, and deletion of ATP6AP2 impairs V-ATPase-dependent vesicular acidification (118, 153, 157). In the absence of ATP6AP2, cells in the renal collecting duct and thick ascending limb showed increased expression of lysosomal proteins and a dramatic increase in the number of autophagic vacuoles. In addition, whole animal studies revealed the presence of metabolic acidosis and a severe urinary-concentrating defect (157). Increased ATP6AP2 expression in the kidneys of diabetic rats and in cultured kidney cells treated with high glucose has also been reported (158–160). Furthermore, an increase in ATP6AP2 expression and activity correlates with the development of insulin resistance in fructose-fed and obese rats, and subsequent ATP6AP2 inhibition improved glucose tolerance in these animals (160, 161). The evolution of insulin resistance is linked to the insulin-dependent activation of the MAPK and transforming growth factor-β1 signaling cascades. Intriguingly, ATP6AP2 activation occurs upstream of these pathways, suggesting that ATP6AP2-induced activation of MAPK signaling may be involved in the pathogenesis of glucose intolerance (153). Additionally, the elevated prorenin/renin levels characteristic of diabetes may contribute to the activation of renal ATP6AP2 and subsequent development of diabetic nephropathy (160). Supporting this hypothesis, treatment of type 1 and type 2 diabetic rats or mice with a peptide inhibitor of ATP6AP2 significantly inhibited the development of diabetic nephropathy (160). Nonetheless, the mechanism underlying this effect and the precise relationship between ATP6AP2 and V-ATPase remain unclear.

## NUTRIENT SENSING

Metabolic homeostasis within cells is controlled by the coordinated actions of AMP-activated protein kinase (AMPK) and the mammalian target of rapamycin complex 1 (mTORC1). AMPK is activated by falling intracellular energy status, which is sensed as an increase in AMP/ATP and ADP/ATP ratios or as a decrease in available glucose (162, 163). Once activated, AMPK increases catabolic processes, thereby generating ATP, while simultaneously inhibiting anabolic pathways, thus reducing cellular energy consumption. Acting in an opposing role to AMPK, mTORC1 integrates signals from available nutrients and growth factors and shifts the metabolic program of the cell from catabolic metabolism to anabolic metabolism by promoting the biosynthesis of necessary components for cell growth (164).

### V-ATPase-mTORC1 Signaling

During mTORC1 activation, under conditions of high amino acid availability, amino acids activate the Rag GTPases, which recruit mTORC1 to the lysosome before its activation (116). mTORC1 activation by amino acids also requires the pentameric Ragulator complex, which acts both as a scaffolding protein anchoring the Rag GTPases (Rags) to the lysosomal membrane and as a GEF toward Rags controlling its activation state (116). Within the past decade the V-ATPase was identified as an upstream component of the mTORC1 regulatory complex involved in the pathway that senses and responds to intralysosomal amino acids. Specifically, the V-ATPase directly interacts with the Ragulator complex in an amino acid-sensitive manner, an interaction that is necessary for the translocation and activation of the mTORC1 complex (116). Of note, the formation of the V-ATPase-Ragulator-Rags complex is also promoted by glucose. Glucose starvation leads to the disassociation of mTORC1 from the V-ATPase-Ragulator-Rags complex and the formation of the V-ATPase-Ragulator-Axin-LKB1-AMPK supercomplex, which will be discussed further below (164, 165).

**Figure 9.**
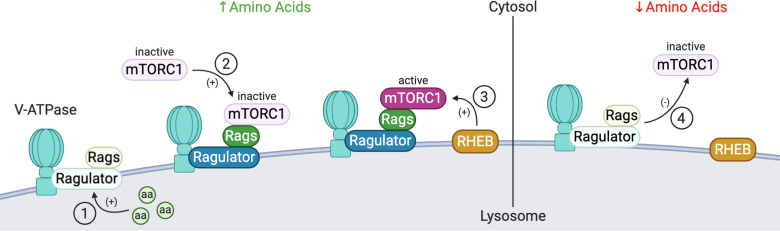
V-ATPase-dependent amino acid signaling. Under conditions of high amino acid (aa) availability (*left*), intralysosomal amino acids activate the Ragulator complex, which acts as a GEF toward the Rag GTPases (Rags), activating them (*1*). The Rag GTPases are anchored to the lysosomal membrane by the Ragulator complex bound to the V-ATPase. Subsequently, inactive mammalian target of rapamycin complex 1 (mTORC1) is recruited to the lysosomal membrane by the active Rag GTPases (2), where it can be activated by the lysosomal Rheb GTPases (*3*). Under low amino acid conditions (*right*), Ragulator GEF activity is inhibited, inactivating Rags, and leading to the disassociation from the lysosome and inactivation of mTORC1 (*4*). Created with BioRender.com.

Intralysosomal amino acids are first sensed by the transmembrane lysosomal protein SLC38A9, which is associated with Ragulator and stimulates Ragulator GEF activity (166). Thus Ragulator activates Rags and promotes a structural rearrangement of the V-ATPase-Ragulator-Rags complex (116). Active Rags recruits mTORC1 to the lysosomal surface where mTORC1 is activated by the Rheb GTPases already present on the lysosomal membrane (116) (Fig. 9). Although V-ATPase rotary motion is required for amino acid sensing and mTORC1 recruitment to occur, the proton gradient generated by the V-ATPase appears to be dispensable, indicating that the V-ATPase is functioning in a signaling role in this context (116). More recently a novel technique for rapidly purifying lysosomes revealed a role for the V-ATPase and mTORC1 signaling in regulating the lysosomal amino acid content. V-ATPase activity appears to be necessary for the export of nonessential amino acids out of the lysosome whereas the export of essential amino acids out of the lysosome seems to require mTORC1 activation (167).

It was shown that amino acid starvation increases V-ATPase assembly on the lysosomal membrane and readdition of amino acids reverses this effect (48). This amino acid-dependent assembly of V-ATPase required activity of AKT serine/threonine kinases (168). Of note, mTORC1 inactivation after amino acid depletion appears to begin before the increase in V-ATPase activity is observed. Futhermore, the newly assembled V-ATPase on the lysosome membrane is catalytically active under low amino acid conditions, which the authors suggest may promote lysosomal degradation under nutrient-poor conditions thereby increasing concentrations of free metabolites (48).

### V-ATPase-AMPK Signaling

**Figure 10.**
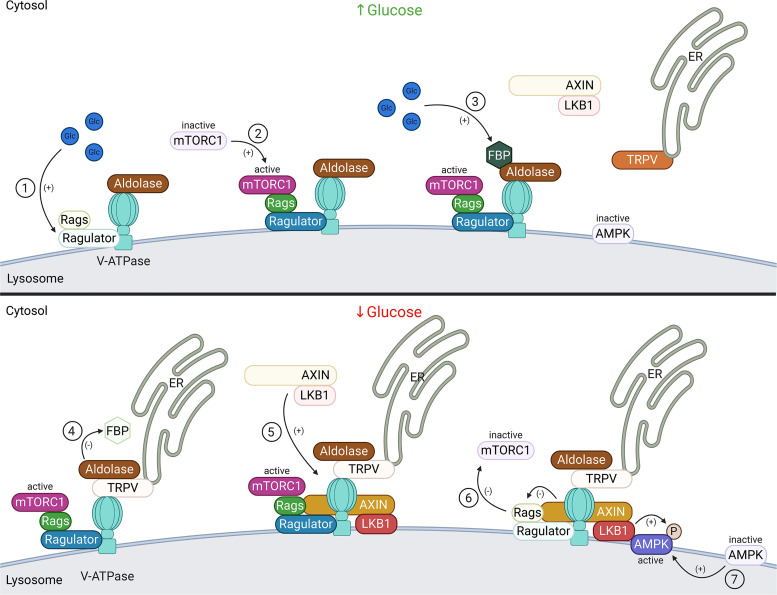
V-ATPase-dependent glucose signaling. When glucose is present (*top*), it activates the Ragulator complex, which acts as a GEF toward the Rag GTPases (Rags), activating them (*1*). The Rag GTPases are anchored to the lysosomal membrane by the Ragulator complex bound to the V-ATPase. Subsequently, mammalian target of rapamycin complex 1 (mTORC1) is recruited to the lysosomal membrane by the active Rag GTPases (*2*), where it can be activated by the lysosomal Rheb GTPases (see Fig. 9). Additionally, when glucose is present, the glycolytic intermediate fructose-1,6-bisphosphate (FBP) binds to the aldolase-V-ATPase complex (*3*). Under conditions of glucose starvation (*bottom*), FBP disassociates from aldolase allowing endoplasmic reticulum (ER)-localized transient receptor potential V-type (TRPV) channels to bind the V-ATPase at the aldolase binding site (*4*). FBP-unoccupied aldolase binds and inhibits TRPV Ca^2+^ channel activity allowing for recruitment of the AXIN-LKB1 complex to the lysosomal membrane (*5*) where it interacts with Ragulator and inhibits its GEF activity toward Rags causing the disassociation from the lysosome and inactivation of mTORC1 (*6*). Furthermore, the V-ATPase, AXIN, and LKB1 recruit AMPK to form a super complex where AMPK is phosphorylated and activated by LKB1 (*7*). Created with BioRender.com.

Further highlighting the importance of the V-ATPase as a key regulator of metabolic signaling pathways, it was subsequently discovered that the V-ATPase-Ragulator-Rags complex also senses low glucose availability. During glucose starvation, the V-ATPase-Ragulator-Rags complex undergoes a conformational change making it accessible to AXIN/LKB1 (164). AXIN directly interacts with the V-ATPase and Ragulator, which recruit the AXIN-LKB1 complex to the lysosomal membrane (164). Intriguingly, pharmacological inhibition of V-ATPase promotes the interaction of AXIN/LKB1 with Ragulator at the lysosome, even under normal glucose conditions (164, 169). Upon recruitment, AXIN inhibits Ragulator GEF activity toward Rags, which leads to the disassociation from the lysosome and inactivation of mTORC1; depletion of AXIN slows the disassociation and decrease in mTORC1 activity from the lysosome after glucose deprivation (164). Surprisingly, AXIN does not seem to participate in the amino acid regulation of mTORC1 (164). After dissociation of mTORC1, AMPK is recruited to the V-ATPase-Ragulator-Axin-LKB1 complex, where it is phosphorylated and activated by LKB1, which promotes the conversion of cellular processes from anabolic to catabolic (164) (Fig. 10).

Intriguingly, the V-ATPase can directly respond to glucose availability in yeast cells. In yeast, the central glycolytic enzyme aldolase physically interacts with three different subunits, a, B, and E, of the V-ATPase in a glucose-dependent manner and regulates V-ATPase assembly and activity (150, 170). Thus, perhaps not surprisingly, glucose-dependent AMPK (de)activation in mammalian cells is also regulated by aldolase, and the glycolytic intermediate and substrate of aldolase, fructose 1,6-bisphospate (FBP) (163). Of note, this regulation of AMPK activation is independent of AMP levels and does not produce the downstream cellular readouts that generally characterize AMPK activation, thus defining a novel mechanism of AMPK activation (163).

When glucose is available, FBP is produced during glycolysis and binds to V-ATPase-associated aldolase. This interaction disrupts the inhibitory binding of AXIN-LKB1 to the V-ATPase-Ragulator-Rags complex at the lysosome, thereby promoting Ragulator GEF activity and Rags activation leading to the recruitment and activation of mTORC1 (163, 169). In the absence of glucose, FBP concentrations decrease leading to FBP disassociation from aldolase. Subsequently, endoplasmic reticulum-localized transient receptor potential V-type (TRPV) channels interfere with the physical interaction of V-ATPase and aldolase by binding directly to the V-ATPase at the aldolase binding site, and FBP-unoccupied aldolase binds to TRPVs and inhibits their Ca^2+^ channel activity (169). The resulting localized decrease in Ca^2+^ concentration allows for TRPV interaction with the lysosomal V-ATPase and formation of the AMPK activation super complex (169) (Fig. 10). Supporting the requirement for TRPV channels in AMPK activation, quadruple knockout of TRPV1-4 significantly inhibited the glucose-dependent formation of the V-ATPase-AXIN-LKB1-AMPK super complex and activation of AMPK (169). Furthermore, AMPK activation upon glucose starvation was severely impaired in the liver of TRPV knockout mice and in *Caenorhabditis elegans* mutants lacking the TRPV orthologues osm-9 and ocr-2 (169, 171).

### Glucose-Dependent Effects on V-ATPase Assembly in Mammalian Cells

There are reports on the effect of glucose on V-ATPase assembly in mammalian cells that appear, at first glance, to be contradictory. One set of studies by the Gluck group (45) shows that low glucose causes disassembly of V-ATPase in cultured cells, in accordance with data from the yeast system. Alternatively, a more recent study by the Forgac group (44) concludes that, in fact, low glucose leads to assembly of the V-ATPase. Importantly, the Gluck team (45) looked at overnight glucose deprivation and examined V-ATPase assembly at both the plasma membrane and intracellular compartments. The Forgac group (44), on the other hand, looked mainly at acute (15 min) glucose starvation and focused on lysosomal assembly. One explanation for these discrepancies could be that the process is dynamic, and while acute glucose starvation leads to assembly on the lysosomal membrane, longer glucose starvation results in overall disassembly of V-ATPase, especially those present at the plasma membrane, to conserve energy.

## SENSORY PERCEPTION

### Auditory Perception

In the inner ear, active proton secretion by the V-ATPase is required to maintain proper pH and ionic concentration (notably high K^+^) of the endolymph bathing the mechanosensory hair cells of the organ of corti (172–175). Indeed, many other acid-base transporters and other ion transporters are also present in portions of the inner ear, including cells lining the endolymphatic sac, indicating a crucial role in hearing. In part because of some similarities in the functional expression of these transporters, drugs and diseases that affect kidney function often have parallel, negative effects in the inner ear (176, 177).

While several epithelial cells of the inner ear express V-ATPase, in the cochlea, the so-called interdental cells are especially V-ATPase-rich and also express the AE1 anion exchanger, thus resembling renal intercalated cells (175). Accordingly, mutations in the a4 or B1 subunit of the V-ATPase, which are known to cause distal renal tubular acidosis, are also associated with sensorineural hearing loss in humans (8, 174, 178, 179) and a4 KO mice are severely hearing impaired and display enlarged endolymphatic fluid compartments (8, 9, 180). The a4 subunit colocalizes with the anion exchanger pendrin at the apical side of epithelial cells lining the endolymphatic sac in the inner ear, similar to what is seen in the type-B intercalated cells in the kidney and mice that lack pendrin have sensorineural deafness, similar to humans with Pendred Syndrome (8, 181–183). Intriguingly, B1 knockout mice were shown to have normal hearing, suggesting a compensatory mechanism of proton transport (184). Possibly, in mice, the B2 subunit can functionally replace the absent B1 subunit in the inner ear, as seen in the A-type intercalated cells in the kidneys and the epididymal clear cells of B1 knockout mice (185, 186).

In addition to the mutations in the B1 and a4 subunit in humans, a disease called dominant-deafness onychodystrophy is caused by a mutation in the ATP6V1B2 subunit gene, resulting in deafness and learning and memory disorders (187). Using a zebrafish model replicating this mutation, it was shown that the V-ATPase can still assemble but that the affinity of some subunits comprising the holoenzyme is lowered. A role of the B2 subunit in other types of cognitive impairment has also been recognized (188, 189).

### Olfactory Perception

Similar to the epithelium of the inner ear, and in other proton-secreting cells, the B1 subunit of the V-ATPase is present at the apical surface of mouse olfactory sustentacular epithelial cells, whereas the B2 subunit is found more evenly distributed throughout the cytoplasm of olfactory epithelial cells and also in olfactory neurons (190, 191). Intriguingly, the B1 subunit is also expressed at the basolateral surface of the olfactory microvillar epithelial cells, which is reminiscent of the expression found in A-type and B-type intercalated cells of the kidney, respectively, and suggests independent functions for the B1-containing holoenzymes in the nose depending on the cell type and membrane localization (190).

Studies in B1-knockout mice showing an increased pH in the neuroepithelial mucosal layer support a role for B1-containing V-ATPase complexes in regulating the pH of the neuroepithelial mucosal layer (191). Further experiments revealed that these mice have decreased innate avoidance and appetitive behaviors, which suggests that maintenance of the proper pH in the mucous layer is important for promoting sensitivity to odorants (191). Furthermore, V-ATPase a4 subunit KO mice have an impaired sense of smell, which suggests that the particular, apically expressed, a4- and B1-containing V-ATPase holoenzymes play a conserved role in maintaining the appropriate pH of the extracellular fluid surrounding acid-secreting sensory cells (9).

It is intriguing that a persistent presentation of COVID-19 coronavirus infection is a loss of smell and taste. It was recently shown that the coronavirus receptor ACE2 is highly expressed on the apical surface of olfactory sustentacular cells (192), precisely the same location as the V-ATPase. Whether the V-ATPase is in some way involved in this sensorial deficit is unknown, but the colocalization of these two enzymes in the same plasma membrane domain, coupled with the known positive effects of V-ATPase on viral entry into cells and in odorant detection, clearly merits further investigation.

### Visual Perception

Although mutations in human V-ATPase are not linked to blindness, a role for the V-ATPase in vision is indicated by studies that identified human patients with osteoporosis-causing mutations in the a3 subunit isoform who also present with visual impairment (193). The V-ATPase is expressed in retinal pigmented epithelia (RPE) in the eyes of *Drosophila*, zebrafish, and rodents, and in the ocular nonpigmented ciliary epithelium (NPE) of rabbits (194–197). Within the NPE in rabbits, the V-ATPase is involved in the production of aqueous humor and inhibition of the V-ATPase using bafilomycin reduced intraocular pressure (IOP) in rabbits suggesting a potential role for increased V-ATPase activity in glaucoma (197). Related to this effect, the carbonic anhydrase inhibitor acetazolamide, which also reduces IOP by suppressing secretion of aqueous humor, has been used as a treatment for glaucoma for over 60 yr (198). Furthermore, in animal models, V-ATPase mutations, or pharmacological inhibition of V-ATPase, decreased autophagy and lysosomal degradation in the RPE leading to the accumulation of undigested proteins and ultimately retinal degeneration (195, 199–202).

Studies in knockout mice showed that phagocytosis and autophagy in RPE cells is regulated by a lysosomal protein, CRYBA1, via the V-ATPase-mTORC1 signaling pathway (203). Furthermore, impaired autophagy in the RPE is associated with human retinopathies including age-related macular degeneration, retinitis pigmentosa, and Usher syndrome type IB, suggesting that V-ATPase disfunction may underlie human retinal pathologies (194–196, 199, 204, 205). The retina and related tissues in the eye have been proposed as accessible targets for various therapeutics, including siRNAs (206); therefore, identifying the molecular components of pathways leading to ocular pathologies in general, but acidification-dependent processes in particular, for diseases such as glaucoma, may be an especially fruitful line of investigation.

### V-ATPases in Insect Sensilla

One of the first reports of V-ATPases localized to the plasma membrane of cells, rather than within endomembranes or vacuolar membranes, was in the epithelial cells lining the midgut of tobacco hornworm (*Manduca sexta*) larvae (207). Unlike in mammalian cells, cationic ion transport in insect epithelia is energized by the V-ATPase (208). This has now been reported not only in the midgut but in many ion-transporting insect epithelia including the Malpighian tubules, salivary glands, labial glands, and sensory sensilla of diverse insects (28, 209). Intriguingly, in the sensory epithelium of *Manduca sexta* and *Antheraea pernyi* antennae, the V-ATPase is expressed in projections protruding from the apical surface of the epithelial cells (209), similar to those seen in activated type-A intercalated cells in the kidney, or in clear cells in the epididymis, which also express plasma membrane V-ATPases (23). This suggests that in addition to a conserved role for certain subunits, such as a4 and B1, as plasma membrane-expressed V-ATPases, the role of plasma membrane V-ATPases in sensory epithelia could be conserved across species. Like hair cells in the inner ear, many of the sensory properties of sensilla depend on the presence of high K^+^ levels in the fluid bathing the apical projections. It is possible that the V-ATPase is required to energize a K^+^ transporter in the same sensilla membrane, as in the insect midgut (28).

## NEURODEGENERATIVE DISEASES

A theme in many common neurodegenerative diseases, including Alzheimer’s disease (AD), Parkinson’s disease (PD), Huntington’s disease, and amyotrophic lateral sclerosis (ALS), is the accumulation of misfolded protein aggregates, which are generated due to failures in the endolysosomal system (118, 210–213). These proteinopathic neurodegenerative diseases are commonly characterized by abnormal V-ATPase function and improper autophagosomal and lysosomal acidification (118, 210, 213). It has long been hypothesized that the V-ATPase misfunction leading to improper acidification of autophagosomes and lysosomes is a major pathogenic factor contributing to the accumulation of protein aggregates that often typifies neurodegenerative diseases. This idea is supported by the fact that many familial neurodegenerative diseases are caused by genetic mutations in V-ATPase subunits or V-ATPase accessory proteins, such as ATP6AP2 (118, 214). Furthermore, most V-ATPase disorders involve central nervous system neurodegeneration and genetic defects in the V-ATPase, which are known to cause improper lysosomal acidification invariably cause neurodegeneration (118, 215). Consistent with these clinical observations, in vitro experiments in *Drosophila* show that a loss-of-function mutation in the V-ATPase leads to impaired proteolysis and age-related neurodegeneration (202). Despite the known involvement of V-ATPase dysfunction in the pathogenesis of neurodegenerative diseases, until recently there has been very little understanding of the mechanisms by which V-ATPase dysfunction contributes to neurodegeneration.

In humans, loss-of-function mutations in presenilin-1/2 (PS1/PS2), ubiquilin-2/4 (UBQLN2/UBQLN4), or leucine-rich repeat kinase 2 are primary causes of familial AD, ALS, and PD, respectively (118, 216–220). Consistently, these mutations are associated with decreased V-ATPase activity, increased lysosomal pH, and impaired autophagy. Intriguingly, the wild-type forms of all of these proteins bind directly to the a1 subunit of the V-ATPase, and the disease-causing mutations abolish this interaction and lead to impaired assembly of a1-containing V-ATPase complexes on the lysosomal membrane (216–220).

In vitro experiments have shown that PS1 is required for proper glycosylation of the V-ATPase, which is necessary for its targeting to and proper acidification of the lysosome (216, 217). Consistent with these findings, cells from AD patients with PS1 mutations have impaired assembly of a1-containing V-ATPase holoenzymes on the lysosomal membrane and more alkaline lysosomes (217). Furthermore, cell culture experiments show that PS1 knockout or PS1/PS2 double knockout cells display lower levels of the a1 subunit on lysosomal membranes, decreased V-ATPase activity, elevated lysosomal pH, enlarged autophagic bodies, and decreased proteolysis similar to what is seen in AD patients (216, 217).

In *Drosophila*, loss of Ubqn/UBQLN results in severe neurodegeneration (218, 220). Additionally, loss of Ubqn/UBQLN leads to increased lysosomal pH, impaired mTORC1 signaling, and decreased autophagic flux in *Drosophila* or cultured mammalian neuronal cells (218, 220). Suggesting that impaired acidification of the lysosome by the V-ATPase is the pathogenic factor, acidic nanoparticles fed to the flies restored lysosomal pH and autophagic flux (220). Furthermore, overexpression of ALS-associated mutants of UBQLN2 led to lower expression of Vha100-1, the *Drosophila* homologue of subunit a1, increased lysosomal pH, and decreased autophagy (218, 220). A recent study using mice and cultured human cells showed that UBQLN2 KO or expression of ALS-causing UBQLN2 mutants reduced expression of the V-ATPase G1 subunit, raised autophagosome pH, and impaired autophagy (221). Furthermore, UBQLN2 was shown to directly interact with the G1 subunit of the V-ATPase, and expression of ALS-associated mutants in UBQLN2 KO cells reduced this interaction. Of note, overexpression of the G1 subunit of the V-ATPase in UBQLN2 KO cells was sufficient to rescue the autophagosome acidification deficit, suggesting that the V-ATPase may be a valid therapeutic target in fighting ALS.

### Role of ATP6AP2 in Neurodegenerative Disease

In the case of the V-ATPase accessory protein ATP6AP2 (also known as PRR, see above), mutations in this gene can lead to PD. Functional studies of stem cell-derived neurons from a patient with ATP6AP2 mutations revealed a decrease in V-ATPase assembly on the lysosomal membrane causing a significant increase in lysosomal pH and impaired protein degradation, ultimately leading to neuronal cell death (222–224). Furthermore, experiments in flies and mice have shown that loss of ATP6AP2 leads to a loss of V-ATPase activity, impairment of autophagy, and severe neurodegeneration mimicking the pathogenesis of ATP6AP2 mutations in humans (222, 223). Mutations in the ceroid lipofuscinosis neuronal 1 gene (CLN1) cause a severe neurodegenerative disease characterized by lysosomal dysfunction (210, 213). *Cln1^−/−^* mice show decreased localization of the a1 subunit to the lysosomal membrane due to deficient palmitoylation of the subunit, which correlates with decreased V-ATPase activity and elevated lysosomal pH (210, 213).

In summary, the impaired assembly of V-ATPase complexes containing the a1 subunit on the lysosomal membrane, leading to increased lysosomal pH and impaired autophagy, contributes to the pathogenesis of a wide variety of genetic neurodegenerative diseases. Consistent with this, loss of the a1 subunit increases the susceptibility of neurons to toxicity caused by peptide aggregates (225).

### Oxidative Stress and Neurodegeneration

Another feature common to the pathology of many neurodegenerative diseases is oxidative stress, although it is not clear whether oxidative stress is a cause of neurodegenerative disease or a secondary effect of neuronal dysfunction (226). Despite this, targeting the oxidative stress associated with neurodegenerative diseases is promising therapeutically as it can ultimately lead to neuronal cell death (227). A family of six proteins containing the highly conserved Tre2/Bub2/Cdc16 (TBC), lysin motif (LysM), and domain catalytic (TLDc) domain (KIAA1609/TLDc1, C20ORF118/TLDc2, Oxr1/TLDc3, Ncoa7/TLDc4, IFI44/TLDc5, and TBC1D24/TLDc6) are involved in the development of the human brain and play a protective role against oxidative stress-related cellular damage (228). Intriguingly, a protein interactome study published by our laboratory identified Oxr1 and Ncoa7, two members of the TLDc family of proteins, as potential binding partners of the V-ATPase (51). Furthermore, loss of V-ATPase activity has been linked to increases in the oxidative stress response in yeast, suggesting that the V-ATPase may play a protective role against oxidative stress (229). Further work is needed to uncover whether the interaction of the V-ATPase with the TLDc family of proteins regulates the oxidative stress response and if this could potentially play a role in the oxidative stress that occurs during neurodegenerative disease.

## VIRAL ENTRY INTO CELLS

A large number of animal viruses affecting humans, including influenza viruses and the coronaviruses responsible for recent global pandemics, are dependent on pH-dependent, receptor-mediated endocytic internalization for their entry into cells (230–232). Once internalized into endosomes, acidification by the V-ATPase triggers conformational changes in the viral proteins, which promotes fusion and release of the replication factors into the cell cytoplasm (233–235). This model is supported by findings that viral infection can often be inhibited by compounds that are known to raise the pH in vesicles of the endomembrane system, such as the weak bases chloroquine and hydroxychloroquine (236–240). Additionally, specific V-ATPase inhibitors prevent the entry of viruses through the endocytic pathway but do not affect infection by viruses that enter the cell by direct fusion with the plasma (233, 241–243). Furthermore, there is evidence in mammalian cell lines and mouse models that the new generation V-ATPase inhibitor diphyllin shows broad spectrum antiviral activity (241, 244).

The principal targets of many antiviral drugs are the viral proteins themselves, but this makes them susceptible to viral mutations conferring resistance, making it increasingly important to identify potential host proteins that influence virulence as alternative drug targets (241, 245–248). Indeed, the use of diphyllin in combination with conventional antiviral drugs conferred increased protection from viral infection in cell culture models of influenza (241). Unfortunately, the importance of the V-ATPase in all aspects of cellular physiology makes global V-ATPase inhibition untenable as a therapeutic. Another promising host protein target is ribonuclease κ (RNASEK), which is required for the replication of many viruses that enter via the acid-dependent endocytic pathway (249, 250). RNASEK is an endosomal protein that associates with the V-ATPase and is required for its function; however, RNASEK is dispensable for general endocytic uptake (249, 250). Thus RNASEK appears to play a singular role in viral entry making it an appealing therapeutic target to prevent infection from prominent human viruses.

### V-ATPase Interaction with SARS Coronavirus

The severe acute respiratory syndrome coronavirus 2 (SARS-CoV-2) outbreak beginning in late 2019 highlighted the importance of finding therapeutics to combat SARS coronaviruses to prepare for, or prevent, future pandemics. A key step in the lifecycle of SARS coronaviruses is the cleavage of the viral genome by its own three C-like proteinase (3CLpro) allowing the release of functional viral replication proteins into the host cell’s cytoplasm. After the first SARS coronavirus outbreak in 2003, the 3CLpro, which is structurally and functionally conserved across many types of viruses, was identified as a promising antiviral target due to its essential role in the replication lifecycle of many viruses and potential to provide broad antiviral protection (251–253).

Intriguingly, a screen of a human lung cDNA library found that the V-ATPase G1 subunit isoform directly interacts with the 3CLpro expressed by the SARS coronaviruses (254). Additionally, the G1 subunit was found to contain a 3CLpro cleavage site and in cell culture experiments was shown to be cleaved by the viral protease. Furthermore, the cleavage of the G1 subunit correlated with a decrease in intracellular pH in 3CLpro-expressing cells, which suggests that this cleavage event may increase V-ATPase activity, thereby promoting the formation of the pH gradient that is necessary for the replication of many viruses (254). Recently, osteoporosis drugs have been developed that specifically target the interaction between the V-ATPase and actin filaments with the purpose of avoiding many of the negative outcomes that occur with broad V-ATPase inhibition (255). Similarly, small molecule inhibitors targeting the interaction of the G1 subunit isoform and the viral 3CLpro could provide specific and broad antiviral protection from viruses that propagate through the endocytic pathway while avoiding the detrimental effects of global V-ATPase inhibition.

### Innate Immune Response to Viral Infection

Another intriguing therapeutic concept is to coopt the body’s own immune defense system to fight viral infections. Interferons (IFNs) combat viral pathogenesis by upregulating the expression of genes whose products act to suppress viral replication (256). One example of this is the IFN-inducible isoform of human nuclear receptor coactivator 7 (Ncoa7), which we recently showed is a V-ATPase regulatory protein (257), and whose expression decreases infectivity of many viruses that enter the cell via endocytosis, including influenza (258). The IFN-inducible isoform of Ncoa7 inhibits the pH-dependent fusion of the viral membrane with the endosomal lipid bilayer, ultimately preventing the release of viral replication proteins into the host cell’s cytoplasm (258). These findings highlight the role of the V-ATPase in mediating the innate immune response and provide a potential avenue for treatment by upregulating the body’s own defenses.

### The V-ATPase as a Target for Viral Therapeutics

As mentioned above, the V-ATPase is directly involved in many aspects of normal cell function, the most well understood of these being the generation of acidic compartments in cells. This decreased pH is, in turn, necessary for processes that include but are not limited to posttranslational modification of proteins and trafficking of intracellular vesicles to their correct destination, including other cellular compartments and the plasma membrane (259). Agents such as hydroxychloroquine are known to have global effects on such intracellular processes. While they may indeed have some efficacy in blocking viral infectivity in some cell types for the reasons outlined earlier, their prolonged use may have deleterious general effects on cell function that manifest in different ways, at different times after administration, and at different sensitivities in various organs. Thus, understanding specific protein interactions and identifying V-ATPase subunits that participate in some but not all of these activities will be a critical part of any therapeutic strategy that focuses on this enzyme complex to target viral infection.

## GRANTS

This work was supported by National Institute of Diabetes and Digestive and Kidney Diseases Institute Grants DK121848 and T32 DK007540.

## DISCLOSURES

No conflicts of interest, financial or otherwise, are declared by the authors.

## AUTHOR CONTRIBUTIONS

A.F.E. and M.M. prepared figures; A.F.E., M.M., and D.B. drafted manuscript; A.F.E., M.M., and D.B. edited and revised manuscript; A.F.E., M.M., and D.B. approved final version of manuscript.
